# Efficacy of noncarbapenem therapy for the treatment of ceftriaxone-resistant Enterobacterales outside the urinary tract

**DOI:** 10.1017/ash.2023.137

**Published:** 2024-01-09

**Authors:** Ethan P. Rausch, Kevin Alby, William Wilson

**Affiliations:** 1 Department of Pharmacy, University of North Carolina Medical Center, Chapel Hill, North Carolina; 2 Department of Pathology and Laboratory Medicine, University of North Carolina Medical Center, Chapel Hill, North Carolina

## Abstract

**Objective:**

To determine the safety of noncarbapenem versus carbapenem antibiotics for treatment of adults with documented infection caused by ceftriaxone-resistant infections outside the urinary tract.

**Design:**

Retrospective cohort of adult patients with a documented infection caused by an extended-spectrum β-lactamase (ESBL)–producing organism isolated between January 2018 and October 2021.

**Setting:**

An academic tertiary-care center.

**Patients:**

Adult patients with a documented infection caused by an ESBL-producing organism outside the urinary tract.

**Methods:**

The primary outcome was a composite of treatment failure defined as 30-day mortality, 30-day readmission, microbiological recurrence, and/or clinical worsening requiring antibiotic change. Secondary outcomes included differentiation of primary composite components and postantibiotic *Clostridioides difficile* infection (CDI).

**Results:**

This study included 130 patients. The primary source of infections were bloodstream (67.7%) and caused by *Escherichia coli* (81.5%). Overall, 101 patients received carbapenem therapy and 29 received noncarbapenem therapy (NCT). NCT was comprised of mainly fluoroquinolones (18 of 29) followed by cefepime (7 of 29). Patients receiving NCT had shorter hospital stays (median, 7 days vs 9 days) and were more often discharged on antibiotics (79.3% vs 50.5%). We did not detect a significant difference in the primary composite outcome of treatment failure for carbapenem (23.8%) versus noncarbapenem treatment (24.2%; *P* = .967). Secondary outcomes included a numerically higher 30-day mortality rate in the noncarbapenem group compared to the carbapenem group: 4 (13.8%) of 29 versus 4 (3.9%) of 101. We did not detect a difference in rates of CDI.

**Conclusion:**

Noncarbapenem therapy may play a role for certain patients with infections caused by ESBL-producing organisms.

In 2019, the Centers for Disease Control and Prevention (CDC) identified extended spectrum β-lactamase (ESBL)–producing Enterobacterales as an urgent threat. Estimations extrapolated from large hospital databases indicated that these infections accounted for ∼200,000 hospitalizations in 2017, placing a large financial burden on many health systems.^1^ This threat has sparked increased interest in the appropriate management of infections caused by ESBL-producing organisms. The MERINO trial, which stopped early due to a significant mortality signal in favor of carbapenems versus piperacillin-tazobactam, established the use of carbapenem antibiotics as the principal treatment of invasive infections caused by ceftriaxone-resistant *Escherichia coli* or *Klebsiella pneumoniae* bacteremia, a common ESBL proxy.^2^ This finding is reflected in the new IDSA guidance document for the management of antimicrobial-resistant gram-negative infections, which supports the use of carbapenems for all infections outside the urinary tract caused by ceftriaxone-resistant *E. coli*, *K. pneumoniae*, or *Proteus mirabilis*, which are organisms likely to produce ESBLs.^3^ With the expanded use of carbapenems based on these recent updates, antimicrobial stewards are faced with a dilemma of maximizing benefits and minimizing risks of long-term sequela from carbapenem use, such as increased cost and development of resistance.^4^

This charge is complicated by the limitations of routine clinical microbiology methodologies to accurately identify ESBL production within organisms. The most common genotypes of ESBL-producing organisms reflect the production of β-lactamase enzymes: CTX-M, TEM, and SHV.^5^ Institutions are often limited by cost and time constraints that do not allow for full genotypic testing. In lieu of genotypic testing, institutions may utilize phenotypic ceftriaxone resistance as a surrogate for ESBL production among Enterobacterales. These organisms would be considered to have in vivo resistance to other broad-spectrum noncarbapenem β-lactams, like cefepime and extended-spectrum β-lactam/β-lactamase inhibitors. This methodology has limitations because these enzymes which lead to ceftriaxone resistance have nuanced differences in how they affect noncarbapenem β-lactams. However, the guidance remains that if utilizing updated cephalosporin breakpoints, no additional ESBL testing is needed, and susceptibility results may be reported as is. The lack of additional reporting or editing of these susceptibility reports may influence the use of antimicrobials that test susceptible, such as extended-spectrum cephalosporins.^6,7^ Additionally, there are likely scenarios in which noncarbapenem β-lactams and other antibiotics may be useful in the management of ceftriaxone-resistant *E. coli*, *K. pneumoniae*, and *P. mirabilis* infections.^5,8,9^ We designed a single-center, retrospective, cohort study to determine the safety of noncarbapenem versus carbapenem antibiotics for the treatment of adults with documented infection caused by ceftriaxone-resistant *E. coli*, *K. pneumoniae*, and *P. mirabilis* infections outside the urinary tract.

## Methods

We conducted a single-center, retrospective, cohort review of adult patients with a documented infection caused by an ESBL-producing organism isolated between January 2018 and October 2021. The study was approved by the local institutional review board (IRB no. 15-0675). We followed STROBE reporting guidelines, which are reflected in the data reporting for this study. Our practice site is a large, academic medical center with an on-site clinical microbiology lab. All isolates of *E. coli*, non-*aerogenes Klebsiella* spp, or *P. mirabilis* demonstrating ceftriaxone-resistance by phenotypic testing were defined as an ESBL-producing organisms. All patients with ESBL-producing organisms during the study period were screened from a report generated with our local electronic medical record (EMR). Admitted adult patients receiving active definitive therapy (defined as ≥48 hours from start of therapy) of either a carbapenem or noncarbapenem alternative for the treatment of ESBL infection outside the urinary tract were included in the study. Noncarbapenem alternatives included all systemic antibiotics other than ertapenem, meropenem, and imipenem-cilistatin. Patients were excluded if they had an organism isolated from a urinary source only, polymicrobial infection, or multiple infections treated within the same encounter, if they expired within 48 hours of the first positive culture. or if antibiotic treatment was withdrawn due to presumed colonization.

The primary outcome was a composite of treatment failure, including 30-day all-cause mortality (from initial culture positivity), 30-day readmission, microbiological recurrence (defined as same site, organism, and susceptibility phenotype within 30-days from last positive culture), and treatment failure (defined as a change of therapy after 72 hours of active antibiotic based on susceptibilities). Secondary outcomes included the individual components of the composite outcome as well as *Clostridioides difficile* infection (CDI) within 30 days of completing therapy or discharge (positive result from a multistep algorithm involving glutamate dehydrogenase plus toxin arbitrated by a nucleic acid amplification test), and a descriptive analysis of microbiological findings. Microbiological findings of interest included distributions of cefepime zone diameters and minimum inhibitory concentrations (MICs) for both cefepime-susceptible and -nonsusceptible organisms. Additionally, we assessed different clinical breakpoint utilizations from major organizations including the European Committee on Antimicrobial Susceptibility Testing (EUCAST) and the Clinical and Laboratory Standards Institute (CLSI) and their impacts on cefepime susceptibility reporting: susceptible (S), ≤1 mg/L; resistant (R) >2 mg/L and S ≤2 mg/L; susceptible dose dependent (SDD), 4–8 mg/L; and R, ≥8 mg/L, respectively.

Throughout the entire study period, our institution utilized cascade reporting of microbiological susceptibilities and did not alter susceptibility reporting, including hiding antibiotic choices, such as cefepime, for likely ESBL-producing Enterobacterales testing resistant to ceftriaxone. Within the study period, the microbiological laboratory transitioned from Kirby-Bauer (KB) disc diffusion susceptibility testing to BD Phoenix automated susceptibility testing (Becton-Dickinson, Franklin Lakes, NJ). Updated CLSI breakpoints were utilized for all antibiotics and organisms, including updated fluoroquinolone breakpoints which occurred during the study period, apart from cefepime. Cefepime breakpoints from EUCAST for Enterobacterales were utilized throughout the entire study period.

Patients receiving definitive carbapenem therapy were compared to those receiving definitive noncarbapenem therapy using the χ^2^ or Fisher exact test for categorical data. Additionally, a multivariate logistic regression was performed including the following covariates: age, source (bloodstream vs nonbloodstream), and receipt of noncarbapenem therapy. Descriptive statistics were used to analyze microbiological data. Analyses were performed in STATA/SE version 16.1 software (StataCorp, College Station, TX), SPSS version 28 software (IBM, Armonk, NY), and Excel version 2016 software (Microsoft, Redmond, WA).

## Results

**Fig. 1.**
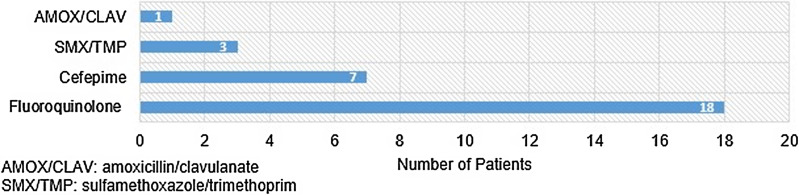
Definitive noncarbapenem therapy.

From January 2018 to October 2021, we screened 347 index encounters. In total, 216 patients were excluded, most commonly due to polymicrobial infection (n = 148, 68.5%), followed by having >1 infection during the admission (n = 57, 26.4%). Additional reasons for exclusion included death within 48 hours (n = 6, 2.8%), isolate from genitourinary tract only (n = 3, 1.4%), and presumed colonization (n = 2, 0.9%). A total of 130 patients were included in the analysis, which comprised 101 carbapenem patients (77.7%) and 29 noncarbapenem patients (22.3%). The average age of the sample population was 63.3 years, and most patients were white and male. *E. coli* was the most commonly isolated organism, accounting for 106 (81.5%) of 130 infections included in our analysis. Bloodstream infections accounted for a majority of the infectious syndromes included with 60 (46.2%) of 130 patients having a primary bloodstream infection, followed by 28 (21.5%) of 130 having secondary bloodstream infections. Additional baseline characteristics are listed in Table 1. Details of the antimicrobial regimens used are listed in Table 1. The most common noncarbapenems used were fluoroquinolones (n = 18, 62.1%), followed by cefepime (n = 7, 24.1%) (Fig. 1). Empiric regimens varied and were used for similar durations of therapy for both carbapenem- and noncarbapenem-treated patients (median, 3 vs 4 days). No statistically significant differences were observed between the 2 groups at baseline. However, we detected some potential clinically significant differences between the groups.

**Table 1.** Baseline Characteristics

Characteristic	Carbapenem (n=101), No. (%)^a^	Noncarbapenem (n=29), No. (%)^a^	Total (N=130), No. (%)^a^
Age, y (SD)	65.8±13.9	54.7±17.8	63.3±15.6
Sex, male	50 (49.5)	16 (55.2)	66 (50.7)
**Race**			
American Indian	3 (2.9)	…	3 (2.3)
Asian	…	1 (3.4)	1 (0.8)
African American	21 (20.8)	10 (34.5)	31 (23.8)
Native Hawaiian	2 (1.98)	…	2 (1.5)
White	61 (60.4)	15 (51.7)	76 (58.5)
Not reported	14 (13.9)	3 (10.3)	17 (13.1)
**Source**			
Blood (1°)	50 (49.5)	10 (34.5)	60 (46.1)
Blood (2°)	26 (25.7)	2 (6.9)	28 (21.5)
Abdomen	9 (8.9)	10 (34.5)	19 (14.6)
Skin and soft-tissue infection	4 (3.9)	1 (3.4)	5 (3.8)
Respiratory	10 (9.9)	5 (17.2)	15 (11.5)
Central nervous system	…	…	…
Bone/joint	1 (0.9)	1 (3.4)	2 (1.5)
Disseminated	1 (0.9)	…	1 (0.8)
**Organism**			
*Escherichia coli*	84 (83.2)	22 (75.9)	106 (81.5)
*Klebsiella pneumoniae*	15 (14.9)	4 (13.8)	19 (14.6)
*Klebsiella. oxytoca*	2 (1.9)	3 (10.3)	5 (3.8)
*Proteus mirabilis*	…	…	…
History of resistant CRO within 1 y	28 (27.7)	3 (10.3)	31 (23.8)
Penicillin allergy	13 (12.9)	4 (13.8)	17 (13.1)
Hospital days of therapy, median d [IQR]	7 [4–10]	4 [2–7]	6 [4–9]
Discharged on therapy	51 (50.5)	23 (79.3)	74 (56.9)
**Empiric antimicrobial regimens**			
**Anti-MRSA**	48 (47.5)	11 (37.9)	59 (45.3)
Vancomycin	44/48 (91.7)	9/11 (31.0)	53/59 (89.8)
Linezolid	3/48 (6.3)	2/11 (6.9)	5/59 (8.5)
Daptomycin	1/48 (2.1)		1/59 (1.7)
**Antibiotic**			
Cefepime	31 (30.7)	13 (44.8)	44 (33.8)
Carbapenem	34 (33.7)	…	34 (26.1)
Piperacillin-tazobactam	17 (16.8)	10 (34.5)	27 (20.8)
Ceftriaxone	13 (12.9)	2 (6.9)	15 (11.5)
Ceftazidime	4 (3.9)	…	4 (3.1)
Fluoroquinolone	2 (1.9)	5 (17.2)	7 (5.4)
Empiric days of therapy, median d [IQR]	3 [2–6]	4 [2–4]	3.5 [2–6]
Hospital LOS, median d [IQR]	9 [7–17]	7 [4–15]	8 [5–14]

Note. SD, standard deviation; CRO, ceftriaxone; MRSA, methicillin-resistant *Staphylococcus aureus*; IQR, interquartile range; LOS, length of stay.

a Units unless otherwise specified.

Comparatively, patients in the carbapenem arm had a numerically higher proportion of primary and secondary bloodstream infections than the noncarbapenem arm (49.5% vs 34.5% and 25.7% and 6.9%, respectively). Additionally, more patients in the carbapenem arm were on carbapenem therapy and MRSA therapy empirically (33.7% vs 0% and 47.5% vs 37.9%, respectively). Patients receiving noncarbapenem therapy were discharged on antibiotics more frequently (79.3% vs 50.5%) and had numerically shorter lengths of stay: 7 days (interquartile range, 4–15) vs 9 days (IQR, 7–17).

We did not detect a statistically significant difference in the primary outcome between carbapenem and noncarbapenem therapy: 24 (23.8%) vs 7 (24.2%) (*P* = .967) (Table 2). The multivariate logistic regression did not reveal increased odds of treatment failure for any potential covariates tested including age, source (bloodstream vs nonbloodstream), and noncarbapenem therapy (Table 3). We also analyzed the primary outcome by its individual components. In those treated with carbapenem therapy, treatment failure was numerically driven by those with a 30-day readmission (n = 19, 18.8%), among whom 4 patients (3.9%) also had microbiological recurrence. The composite primary outcome for those receiving noncarbapenem therapy was nearly split between 30-day all-cause mortality and 30-day readmission: n = 4 (13.8%) and n = 3 (10.3%). No cases of treatment failure, per our definition, were noted. Additionally, we did not detect a significant difference in CDIs.

**Table 2.** Treatment Outcomes

Outcome	Carbapenem (n=101), No. (%)	Noncarbapenem (N=29), No. (%)	Total (N=130), No. (%)
Primary composite outcome	24 (23.8)^a^	7 (24.2)^a^	31 (23.8)
30-d readmission	19 (18.8)	3 (10.3)	22 (16.9)
30-d mortality	4 (3.9)	4 (13.8)	8 (6.2)
Microbiological recurrence	4 (3.9)	…	4 (3.1)
Treatment failure at 72 h	…	…	…
*Clostridioides difficile* infection	2 (1.9)	0	2 (1.5)

a *P* = .967.

**Table 3.** Multivariate Logistic Regression

Factor	Odds Ratio	95% Confidence Interval
Age	1.003	0.976–1.031
Source	0.736	0.341–1.590
Noncarbapenem use	0.937	0.331–2.651

We analyzed bloodstream infections, including both primary and secondary. Overall, 76 patients were treated with carbapenems versus 12 treated with noncarbapenems. The primary composite outcome of treatment failure was similar for those treated with carbapenems and noncarbapenems: 17 (22.4%) of 76 versus 3 (25.0%) of 12, respectively. Mortality was numerically higher in the noncarbapenem group (n = 1 of 12; 8.3%) compared to the carbapenem group (n = 3 of 76; 3.9%), similar to the entire study population, however this finding is limited by sample size. Readmission and microbiological recurrence were otherwise similar (Supplementary Materials). Additionally, the same trends occurred for nonbloodstream infections, with a greater numerical disparity in the mortality rate for those treated with carbapenems versus noncarbapenems (4.0% vs 17.6%).

All 347 index encounters were analyzed for microbiological outcomes regarding cefepime susceptibility. Kirby-Bauer susceptibility methods were utilized on 275 isolates with a median zone diameter of 17 mm (IQR, 12–21). BD Phoenix automated susceptibility testing was utilized on 72 isolates, with a median minimum inhibitory concentration (MIC) of 16 mg/L (IQR, 4–16). Using EUCAST cefepime susceptibility breakpoints, 26 isolates (7.5%) tested susceptible (17 of 275 by Kirby-Bauer and 9 of 72 by BD Phoenix) compared to 45 isolates (13.0%) per CLSI susceptibility breakpoints (32 of 275 by Kirby-Bauer and 13 of 72 by BD Phoenix). Of the 29 patients included in the noncarbapenem therapy group, 7 tested susceptible to cefepime (5 by Kirby-Bauer and 2 by BD Phoenix), all of whom received cefepime therapy. Additional details of microbiological outcomes are provided in the Supplementary Material.

## Discussion

We did not detect a statistically significant difference in a composite end point of treatment failure when comparing carbapenem versus noncarbapenem treatment of ceftriaxone-resistant Enterobacterales outside the urinary tract. Additionally, multivariate logistic regression did not reveal increased odds of treatment failure with noncarbapenem therapy, age, or source of infection. These results were consistent overall across bloodstream and nonbloodstream infectious sources.

Interestingly, patients who received noncarbapenem therapy were more often discharged on antimicrobials and had shorter hospital lengths of stay, numerically. These differences, although not statistically significant, may be clinically significant. We hypothesize that earlier discharge on antimicrobials was likely influenced by the availability of oral alternatives, such as fluoroquinolones or trimethoprim-sulfamethoxazole, when using a noncarbapenem approach.

In the noncarbapenem arm, 4 (80%) of 5 patients who started empirically on fluoroquinolone therapy completed their treatment course with a fluoroquinolone, none of whom met the primary outcomes. Although these numbers were small, they are encouraging and warrant further study. If validated, these findings may allow for continuation of active noncarbapenem empiric therapy if it is later determined that the isolate is ceftriaxone resistant and clinically the patient has improved. Ultimately, this change may prevent additional antibiotic exposure, which may reduce CDI risk and unnecessary changes in a patient’s regimen.^10^

These findings are advantageous from a stewardship perspective given the ability of oral therapy to decrease the length of hospital stay and the amount of antimicrobial exposure. Oral therapy also avoids the need for intravenous access, which can lead to increased adverse events as well as higher total treatment costs.^11^ Continued analysis will help us understand how we can best maximize these advantages.

Our data add to recent evidence that noncarbapenem therapy may have a certain role in the management of ceftriaxone-resistant Enterobacterales infections.^8,9^ Anderson et al^9^ conducted a similar retrospective cohort comparing noncarbapenem β-lactams to carbapenems for the management of ceftriaxone-resistant Enterobacterales infections; however, their analysis was confined to infections of the urinary tract. They included 492 patients (35% with noncarbapenem β-lactam therapy vs 65% with carbapenem therapy). No statistically significant differences were detected between the groups regarding positive clinical response and mortality. Mortality was low compared to other studies involving ESBL-producing organisms, although this finding was likely influenced by the source of infection being the urinary tract.^2^ Their analysis was limited by the extremely favorable conditions of the urinary tract for treatment success, even in the setting of suboptimal treatment.^9^ Regardless, these data highlight the possibility for a carbapenem alternative approach in some patients. Our data extend the generalizability such findings in that we included only infections outside the urinary tract. Additionally, they utilized a surrogate marker of ESBL production similar to our study, which defined ESBL-producing organisms as those with phenotypic ceftriaxone resistance on susceptibility reporting.

The utility of ceftriaxone resistance as a surrogate marker and debate regarding the value of alternative genotypic testing for ESBL-producing organisms have recently come into question.^12,13^ One such area of debate is the use of cefepime for ceftriaxone-resistant Enterobacterales isolates given its expanded stability to CTX-M compared to ceftriaxone and the overall mixed clinical efficacy data.^8,14^ Our microbiology laboratory does not alter cefepime susceptibility reporting based on ceftriaxone resistance, meaning that cefepime susceptibility is reported for all ceftriaxone-resistant Enterobacterales. We also use a more stringent cefepime MIC breakpoint of 1 mg/L, according to EUCAST recommendations. EUCAST has set this lower breakpoint as a surrogate to rule out ESBL production because cefepime MICs are likely to be higher in ESBL-producing organisms.^7^

We hypothesize that this reporting likely influenced treatment choices because almost 2-fold more isolates would have been reported as susceptible at the CLSI breakpoint of 2 mg/L. All 7 patients in the noncarbapenem cohort that had an isolate that tested susceptible to cefepime were treated with cefepime, and 4 of these patients experienced the primary composite outcome of treatment failure (Supplementary Table 3). Dosing was not collected for these patients, but in general, high-dose cefepime 2 grams every 8 hours is utilized for the vast majority of patients at our institution. These findings are not conclusive and are limited by small sample size but may caution the use of cefepime even despite use of high dose cefepime and more restrictive cefepime Enterobacterales breakpoints. Additionally, this study highlights that microbiologic reporting of these isolates may influence antimicrobial use, especially in the case of not having a microbiologic comment denoting possible ESBL-producing organism in lieu of genotypic data.

Given the retrospective nature of our study, it had several limitations. A major limitation of our study is confounding by indication because it is likely that carbapenems were used in more severely ill patients, which we can see in the arithmetical difference in source of infection for the cohorts. No statistical control was performed outside a multivariate analysis conducted to attempt to control for some high-risk confounders (ie, age and source of infection). Another limitation is that the inclusion–exclusion scheme utilized may have excluded early death or treatment failure for some patients, which may have led to underestimates of overall mortality or treatment failure in the study. A total of 6 patients were excluded from our analysis based on this inclusion–exclusion scheme.

Additionally, the noncarbapenem arm had numerically higher all-cause mortality; however, a review of the mortality cases in the noncarbapenem arm revealed that 2 of the 4 patients had noninfectious-related mortality outcomes. One patient transitioned to comfort care after discharge and completion of antibiotic therapy and the other died from complete respiratory failure secondary to status epilepticus. The other 2 deaths were likely related to infection.

Surprisingly, the largest difference in mortality for patients treated with carbapenems versus noncarbapenems was seen in nonbloodstream infections. This finding may have been influenced by the type of infection being primarily respiratory (likely hospital or ventilator-acquired pneumonias) or intra-abdominal infections. Overall, we caution the conclusiveness of these findings given the small sample size in our study.

Our data expand on noncarbapenem use for invasive infections caused by ceftriaxone-resistant Enterobacterales. Alternative noncarbapenem use is attractive from an antimicrobial stewardship standpoint, and our data support its continued exploration. We detected a preference to use carbapenems for bloodstream infections, which was likely influenced by the findings of the MERINO randomized control trial. The mortality benefit concluded in MERINO cannot be overlooked and supports the use of carbapenems for most invasive infections caused by ESBL-producing organisms. Our data indicate that a certain subset of patients may benefit from noncarbapenem antimicrobial use. Such patients may include those in whom enteral therapy would be preferred, those who do not have access to outpatient parenteral antimicrobial therapy, those who have clinically improved on empiric noncarbapenem therapy, or those unable to remain hospitalized to complete their course.

In conclusion, our findings call into question the need for carbapenem therapy for all patients infected with ceftriaxone-resistant Enterobacterales. Our findings also support ongoing investigations into populations most likely to benefit from a noncarbapenem approach as well as analysis of any agent that may be preferred (ie, fluoroquinolones vs some alternative). Long-term effects of such an approach may have a lasting impact on the outcomes of our patients affected by ESBL-producing organisms.
